# The *BIRC6* gene as a novel target for therapy of prostate cancer: dual targeting of inhibitors of apoptosis

**DOI:** 10.18632/oncotarget.2229

**Published:** 2014-07-17

**Authors:** Sze Ue Iris Luk, Hui Xue, Hongwei Cheng, Dong Lin, Peter W. Gout, Ladan Fazli, Colin C. Collins, Martin E. Gleave, Yuzhuo Wang

**Affiliations:** ^1^ The Vancouver Prostate Centre, Vancouver General Hospital and Department of Urologic Sciences, the University of British Columbia, Vancouver, BC, Canada; ^2^ Department of Experimental Therapeutics, BC Cancer Agency, Vancouver, BC, Canada

**Keywords:** BIRC6, IAP, cIAP1, survivin, ASO, CRPC

## Abstract

Treatment resistance, the major challenge in the management of advanced prostate cancer, is in part based on resistance to apoptosis. The Inhibitor of Apoptosis (IAP) protein family is thought to play key roles in survival and drug resistance of cancer via inhibition of apoptosis. Of the IAP family members, cIAP1, cIAP2, XIAP and survivin are known to be up-regulated in prostate cancer. BIRC6, a much less studied IAP member, was recently shown to be elevated in castration-resistant prostate cancer (CRPC). In the present study, we showed a correlation between elevated BIRC6 expression in clinical prostate cancer specimens and poor patient prognostic factors, as well as co-upregulation of certain IAP members. In view of this, we designed antisense oligonucleotides that simultaneously target BIRC6 and another co-upregulated IAP member (dASOs). Two dASOs, targeting BIRC6+cIAP1 and BIRC6+survivin, showed substantial inhibition of CRPC cell proliferation, exceeding that obtained with single BIRC6 targeting. The growth inhibition was associated with increased apoptosis, cell cycle arrest and suppression of NFkB activation. Moreover, treatment with either dASO led to significantly lower viable tumor volume *in vivo*, without major host toxicity. This study shows that BIRC6-based dual IAP-targeting ASOs represent potential novel therapeutic agents against advanced prostate cancer.

## INTRODUCTION

Prostate cancer is the most common non-cutaneous cancer and the second leading cause of cancer-related deaths for males in the Western world [1]. Prostate cancers are initially androgen-dependent, and while androgen deprivation therapy (ADT) can induce marked tumor regression, resistance to ADT inevitably emerges, leading to castration-resistant prostate cancer (CRPC). The current standard care for treating CRPC is systemic, docetaxel-based chemotherapy, increasing the overall survival of patients by about 2 months compared to mitoxantrone-based therapy [2, 3]. Recently, sipuleucel-T, cabazitaxel, abiraterone, MDV3100 and Radium-223 have shown more prolonged overall survival benefit and are approved by the FDA for treatment of the disease [4]. However, none of these drugs therapies are curative; they incrementally improve overall survival. Clearly, establishment of more effective therapeutic targets and drugs, specifically those targeting the molecular drivers of metastatic CRPC, is of critical importance for improved disease management and patient survival [5].

Apoptosis, a cell death-inducing process important in the regulation of cell numbers in normal tissues, can be triggered by a variety of death signals from both extracellular and intracellular origins, and involves activation of caspases (intracellular cysteine proteases) that mediate the execution of apoptosis [6]. Human cancers are characterized by resistance to apoptosis, intrinsic or acquired, considered to be a key factor underlying resistance to therapeutic intervention, and promising new strategies have been developed based on drug-induced apoptosis [7]. The treatment resistance of CRPC is thought to be based on increased resistance to apoptosis and may be addressed by targeting anti-apoptotic genes and their products [8].

The Inhibitors of Apoptosis (IAP) form a family of functionally and structurally related proteins that have a major role in cell death regulation. They act as endogenous apoptosis inhibitors by binding to caspases, thereby suppressing apoptosis initiation. The human IAP family consists of 8 members that are characterized by the presence of 1 to 3 baculovirus inhibitor of apoptosis repeat (BIR) motifs that are involved in the binding of IAPs to caspases. There is increasing evidence that IAPs also affect other cellular processes, such as ubiquitin-dependent signalling events that activate nuclear factor κB (NFκB) transcription factors, which in turn drive the expression of genes important in cellular processes such as cell survival [9]. Due to their ability to control cell death and elevated expression in a variety of cancer cell types, IAP proteins are attractive targets for the development of novel anti-cancer treatments [10]. Four IAP members, i.e. XIAP, survivin, cIAP1 and cIAP2, have been reported to be up-regulated in prostate cancer [11]. Survivin in particular is promising as a potential therapeutic target for the disease [12, 13].

The *BIRC6* gene (*BRUCE/APOLLON*) encodes a 528 kDa protein in mammals, consisting of a single N-terminal BIR domain and a C-terminal ubiquitin-conjugating (UBC) domain; the latter has chimeric E2/E3 ubiquitin ligase activity as well as anti-apoptotic activity [14]. Through its BIR domain, BIRC6 protein can bind to active caspases, including caspases-3, 6, 7 and 9 and such interactions have been shown to underlie its ability to inhibit the caspase cascade and ultimately apoptosis [14]. Through its UBC domain, BIRC6 facilitates proteasomal degradation of pro-apoptotic proteins, including caspase-9 [15], SMAC/DIABLO [15, 16] and HTRA2/OMI [14, 17]. Elevated expression of BIRC6 has been found in a variety of cancers, i.e. childhood *de novo* acute myeloid leukemia [18], colorectal cancer [19], neuroblastoma [14, 20], melanoma [21] and non-small cell lung cancer [22]. Furthermore, BIRC6 has been implicated in maintaining resistance against cell death stimuli [23, 24]. In contrast to other IAPs, BIRC6 has been shown to have a cytoprotective role, essential for survival of mammalian cells [15, 25]. BIRC6 is also known for its essential role in regulating cytokinesis, a final event of cell division [26]. The dual roles of BIRC6 in cell death and division processes resemble those of survivin, and render it a promising target for therapy of a variety of cancers [27].

We recently showed elevated expression of BIRC6 in prostate cancer cell lines and clinical specimens, and found that increased BIRC6 expression was associated with Gleason score 6-8 prostate cancers and CRPC, suggesting a role for BIRC6 in prostate cancer progression and castration resistance [28]. In the present study, we established, using a larger cohort of clinical prostate cancer samples, a correlation between elevated BIRC6 expression and advanced prostate cancer - evidence supporting a role for BIRC6 in the malignant progression of the disease. We designed antisense oligonucleotides (ASOs) that simultaneously target BIRC6 and an additional IAP to achieve maximal anti-tumor activity, as elevated expression in prostate cancer has also been reported for other IAPs such as survivin and cIAP1. Promising results have been found using *in vitro* and *in vivo* models.

## RESULTS

### Elevated BIRC6 protein expression is associated with poor prognostic factors in prostate cancer

**Figure 1:**
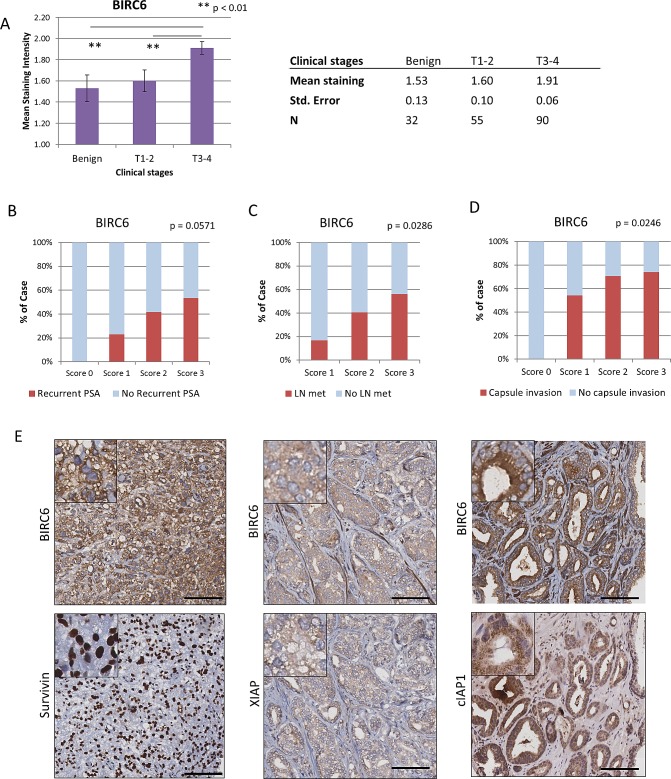
Elevated BIRC6 expression is associated with advanced stages of prostate cancer: co-upregulation of other IAP members (A) Correlation of immunohistochemical staining intensity of BIRC6 and clinical (T) stages of prostate cancer (mean staining intensity ± S.E.M.). (B-D) Correlation of BIRC6 immunohistochemical staining intensity with the absence and presence of poor prognostic factors, such as recurrence of PSA, lymph node metastasis and prostatic capsule invasion. The statistical significance of positive trends was determined by the Chi square test for trend. (E) Representative images of correlated expressions between BIRC6 and survivin, XIAP and cIAP1. 20x magnification, scale bar, 100 μm.

We examined whether various clinical parameters of prostate cancer, i.e. clinical T stage, PSA recurrence, lymph node metastasis and prostatic capsule invasion, were associated with changes in BIRC6 protein expression. Immunohistochemical staining of BIRC6 in prostate cancer tissue arrays showed that BIRC6 expression was elevated in tumors at more advanced clinical stages, i.e. expression of BIRC6 was significantly higher in T3-4 stage tumors than in T1-2 stage tumors or benign prostate (mean intensity ± S.E.: 1.91 ± 0.06, 1.60 ± 0.10 and 1.53 ± 0.13, respectively; Benign to T3-4, p = 0.0032; T1-2 to T3-4, p = 0.0059; Student's t test) (Fig. 1A). Elevated BIRC6 expression also correlated positively with poor prognostic factors such as PSA recurrence (Fig. 1B), lymph node metastasis (Fig. 1C) and prostatic capsule invasion (Fig. 1D) (p = 0.0571, 0.0286 and 0.0246, respectively, Chi square test for trend), indicative of its association with more advanced prostate cancer. The expression of survivin was also elevated in prostate cancer specimens (p = 0.004, Benign to T3-4), and correlated similar to BIRC6 with the above poor prognostic factors (p = 0.0167, PSA recurrence; p = 0.028, capsule invasion; p = 0.006, lymph node metastasis). Elevated XIAP expression was observed in prostate cancer and poor prognostic factors; however, statistical significance was not reached. No correlation was seen in cIAP1 (Fig. S2). Taken together, the data indicate that BIRC6, like survivin, may play a role in prostate cancer progression.

### Positive correlation between expressions of BIRC6 and other IAP members in human prostate cancer

To establish whether there was a correlation between increases in the expression of BIRC6 in prostate cancer and those of other IAP members, the IHC expression profiles of BIRC6, XIAP, survivin and cIAP1 in individual clinical prostate samples (including benign tissue, primary cancer and CRPC) were analyzed for correlations by the Spearman's rank correlation test using GraphPad 4 software. The Spearman r coefficients for the BIRC6 – survivin and BIRC6 – XIAP combinations were 0.3987 and 0.6025, respectively (p < 0.0001), indicating positive correlations between BIRC6 and survivin, and between BIRC6 and XIAP. A weak, but significant, positive correlation was observed for the BIRC6 – cIAP1 combination, with a Spearman r coefficient of 0.194 (p = 0.0072). The positive correlations between the expressions of BIRC6 and the other IAPs were visualized by representative IHC stained images of matched patients’ samples (Fig. 1E).

### Dual IAP-targeting antisense oligonucleotides suppress prostate cancer cell proliferation

**Figure 2:**
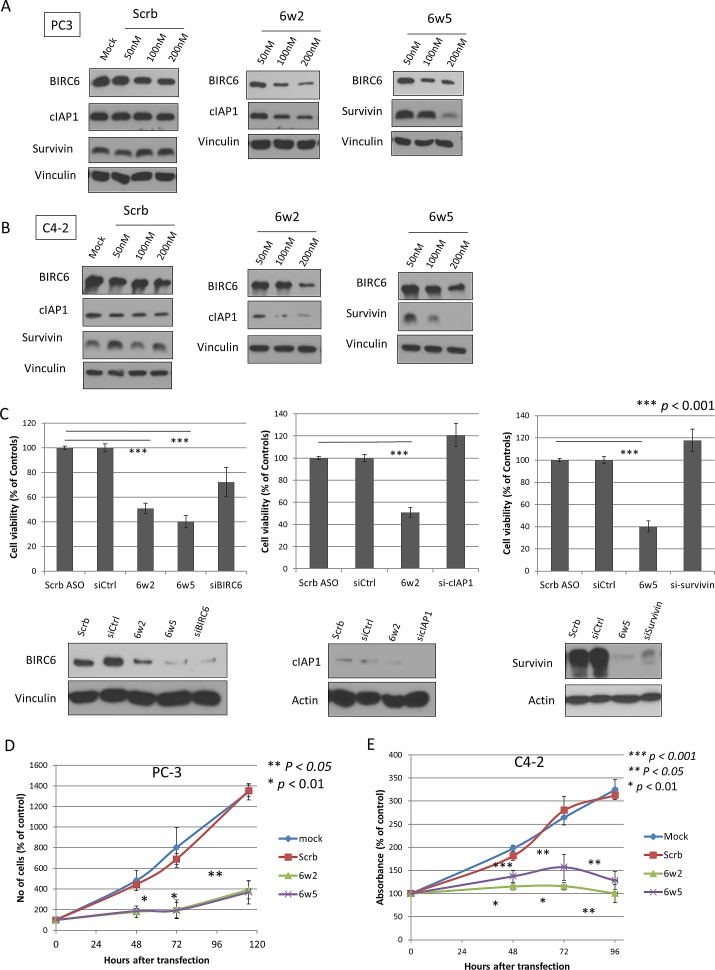
Dual IAP-targeting ASOs knockdown BIRC6, cIAP1 or survivin proteins and lead to marked suppression of CRPC cell proliferation (A-B) Western blotting showing protein levels of BIRC6, cIAP1 and survivin in two CRPC cell lines (A) PC-3 cells and (B) C4-2 cells transfected with Mock or increasing dosages of scrambled ASO (Scrb), dASOs 6w2 and 6w5 for 72 hr. (C) Comparison of dual IAP targeting and single IAP-targeting. Cell viability of PC-3 cells transfected with dASOs 6w2, 6w5 and siRNA-targeting BIRC6, cIAP1 or survivin, was determined by MTS assay at 72 hr after transfection. Cell viabilities of ASO- and siRNA-treated cells were normalized with corresponding Scrb ASO and siRNA controls. Error bars represent mean percentage cell viability ± S.D. Western blotting of 3 IAPs showing comparable amounts of reduced protein expression obtained with dASO and siRNA single IAP-targeting. (D) Proliferation of PC-3 cells transfected with mock, Scrb ASO, dASOs 6w2 and 6w5. Error bars represent mean cell number ± S.D. (E) MTS viability assay of C4-2 cells treated with dASOs.

As BIRC2 (cIAP1), BIRC4 (XIAP) and BIRC5 (survivin) tended to be co-upregulated in prostate cancer in addition to BIRC6, simultaneous targeting of BIRC6 plus one of these IAP members was more likely to give superior anti-cancer effects. Accordingly, dual-targeting antisense oligonucleotides (dASOs), specifically targeting combinations of BIRC6 with each of the other three IAPs, i.e. 6w2, 6w4, 6w5, were tested for reduction of BIRC6 protein expression. As shown in Supplementary Fig. S3, only 6w2 and 6w5-1 markedly reduced BIRC6 protein levels in prostate cancer cells. The effects of these two dASOs on BIRC6, BIRC2 and BIRC5 protein levels were then tested by treating PC-3 and C4-2 cells with increasing doses of the dASOs. As shown in Figures 2A and 2B, treatment with dASO 6w2 (100 and 200 nM) resulted in marked, dose-dependent reductions in both BIRC6 and cIAP1 protein expression; similarly dASO 6w5-1 (100 and 200 nM) (in the following text referred to as 6w5), led to marked reductions in both BIRC6 and survivin protein expressions. A time course experiment showed that treatment of PC-3 cells with dASOs 6w2 and 6w5 resulted in a marked reduction in BIRC6 protein expression after 48 hours of transfection, whereas reduction in cIAP1 and survivin protein expressions by these dASOs started at 72 hours after transfection (Fig. S4).

The anti-cancer effects obtained by single and dual targeting of IAPs were compared. At a comparable degree of silencing of BIRC6, cIAP1 and survivin, knockdown of each IAP alone by siRNA did not result in marked reduction in viable PC-3 cell numbers compared to the mock control (27.8%, -20.8% and -17.7%, respectively). However, simultaneous silencing of BIRC6+cIAP1 and BIRC6+survivin by 6w2 and 6w5, respectively, led to marked reductions in the number of viable cells (49.1% and 59.8% of suppression, respectively, *p* <0.001). Since different silencing methodologies were used, i.e. siRNA and ASO, that presumably work via different mechanisms [29], the viabilities of cells treated with either method were normalized using the cell viabilities obtained with the corresponding, non-targeting controls (Fig. 2C).

The activities of 6w2 and 6w5 were more closely examined in time course studies using cell proliferation/viability assays. Dual silencing of BIRC6 + cIAP1 in PC-3 cell cultures by 6w2 effectively suppressed cell proliferation at 48, 72 and 115 hours by 77.0%, 82.4% and 76.7%, respectively, compared to Scrambled (Scrb) control (p<0.05). Similarly, silencing of BIRC6 + survivin by 6w5 resulted in 74.7%, 84.1% and 78.5% growth inhibition compared to Scrb at the same time points (p < 0.05) (Fig. 2D). A consistent growth-inhibitory trend was also observed using C4-2 cells and viability assays. The growth suppressions obtained with 6w2, compared to Scrb, at 48, 72 and 96 hours were 81.2% (p <0.001), 91.1% (p <0.01) and 99.9% (p <0.01), respectively, and those obtained with 6w5, compared to Scrb at 48, 72 and 96 hours were 54.0% (p <0.05), 68.3% (p <0.05) and 86.8% (p <0.01), respectively (Fig. 2E). Reductions in BIRC6 protein expression were also observed in cells treated with 100 and 200 nM scrambled ASO, but to a lower extent than obtained with the targeting ASOs. For further studies, PC-3 cells were selected due to their higher sensitivity to BIRC6 silencing.

### dASOs 6w2 and 6w5 induce apoptosis, cell cycle arrest and suppress NFkB activation

**Figure 3:**
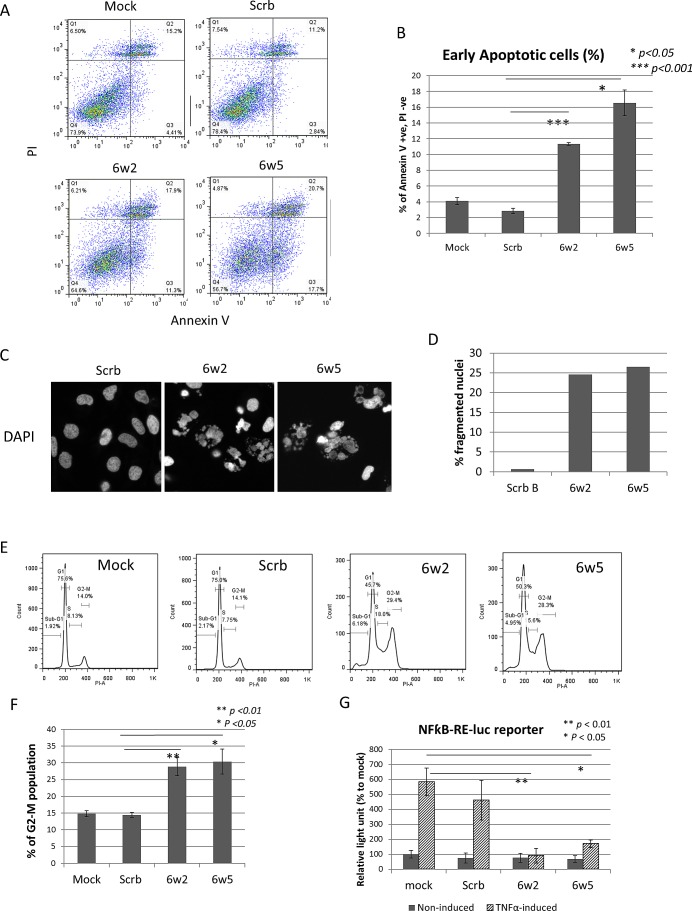
dASOs 6w2 and 6w5 induce apoptosis, cell cycle arrest and abolish NFkB signaling (A-B) Annexin V assay of PC-3 cells treated with dASOs 6w2 and 6w5 for 72 hr. (A) FACS plot showing cells under early apoptosis as identified by Annexin V +, propidium iodide (PI) -. (B) Mean percentage of early apoptotic cells from Annexin V assay. Error bars represent mean ± S.D. (C-D) DAPI staining of dASO-treated cells. (C) Representative images of PC-3 cells stained with DAPI after 72 hr of dASO treatment; apoptotic cells were identified by fragmented nuclei. (D) Quantification of cells undergoing apoptosis: percentage of fragmented nuclei. (E) Cell cycle distribution of PC-3 cells treated with ASOs for 72 hr as determined by PI staining. (F) Percentage of cells at the G2-M phase. (G) NFkB transcription activation was examined using a NFkB dual luciferase reporter assay. PC-3 cells were co-transfected with dASOs, NFkB-responsive firefly luciferase and Renilla luciferase plasmid. Luciferase activity was measured at 48 hr after transfection with prior induction by TNF-α treatment.

To understand the cause of growth-inhibition produced by dASOs, apoptosis induction was first investigated. PC-3 cells were incubated with dASOs 6w2 and 6w5 for 72 hours and then subjected to Annexin V and PI staining and FACS analysis to determine the amount of early apoptotic cells generated. FACS analysis showed that the treatments led to apoptosis of 11.3% and 16.6% obtained with 6w2 and 6w5, respectively, compared to 2.8% obtained with Scrb ASO (p = 6.68 × 10^−5^ and 0.047, respectively) (Fig. 3A, B). In addition, PC-3 cells treated with dASOs 6w2 and 6w5 for 72 hours were stained with DAPI and the numbers of fragmented nuclei (a key indicator of apoptosis) were determined under a fluorescent microscope. The percentage of cells containing apoptotic nuclei was 24.6% and 26.5% for 6w2- and 6w5-treated cells, respectively, in contrast to 0.64% for Scrb ASO-treated cells (Fig. 3C, D). FACS analysis of PI-stained PC-3 cells showed that dASO 6w2 and 6w5 treatments were associated with significant increases in the G2-M phase population [28.9% for 6w2 (p=0.008) and 30.4% for 6w5 (p=0.015)], compared to the Scrb control (14.4%) and mock control (14.8%) (Fig. 3E, F). Increases in S phase population were also observed in both treated groups.

In view of a close link between IAPs and the NFkB pathway [30, 31], the effects of dASOs 6w2 and 6w5 on NFkB transactivation in PC-3 cells were examined using a dual luciferase reporter assay under TNFα-induced and non-induced conditions. The TNFα-induced NFkB activation was markedly suppressed in dASO 6w2-treated cells compared to cells treated with Mock (97.0%, % suppression to mock, p = 0.003), whereas NFkB activation was 20.2% suppressed by Scrb ASO compared to Mock. A marked suppression of NFkB activation was also observed in dASO 6w5-treated cells (79.0%, % of suppression to mock, p = 0.011) (Fig. 3G). Furthermore, siRNA silencing of BIRC6 did not reduce TNFα-induced NFkB activation, in contrast to silencing of cIAP1 (p = 0.029 and 0.012 to Mock and siCtrl, respectively), indicating that the dASO-induced inhibition of NFkB transactivation was not caused by reduction of BIRC6 protein expression (Fig. S5).

Taken together, the results demonstrate that the growth suppression of dASO 6w2- and 6w5-treated PC-3 cells was associated with apoptosis induction, G2-M phase arrest and repression of NFkB promoter activation, highlighting the multifaceted action of both dASOs.

### dASOs suppress PC-3 xenograft growth

**Figure 4:**
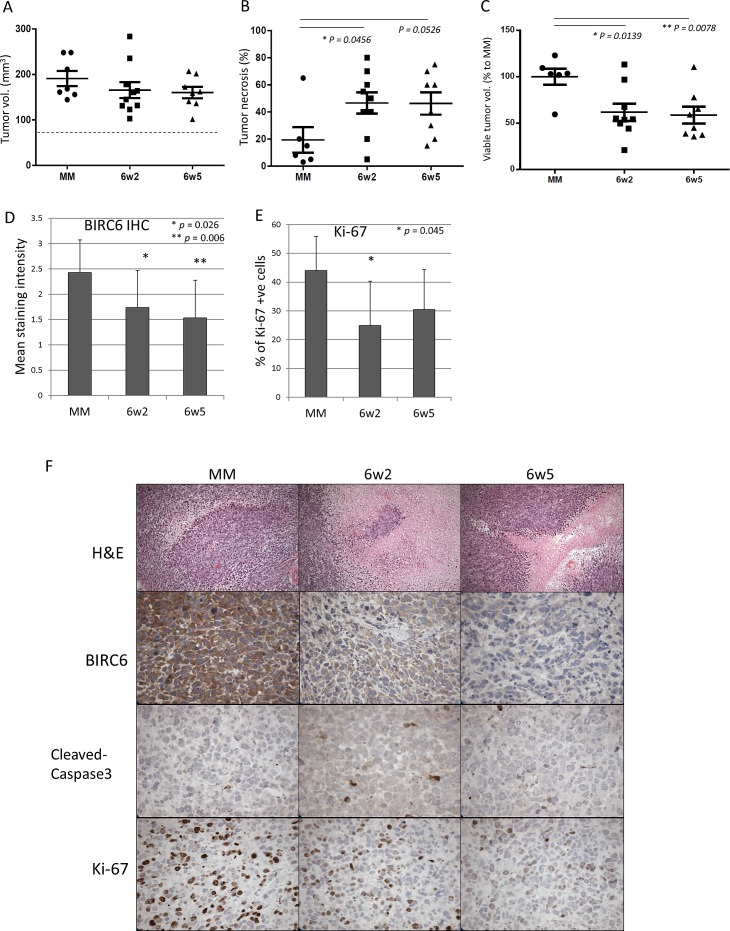
Treatments with dASOs resulted in significant lower viable tumor volume (A) Total tumor volumes at the end of treatment (day 15). NOD-SCID mice with established PC-3 subcutaneous xenografts were treated with control, 6w2 and 6w5 dASOs for 15 consecutive days and tumors were harvested at the end of treatment. Dash line refers to mean tumor volumes at day 0 (before treatment, 78 mm^3^). (B) Percentage of tumor necrosis at harvest determined by H&E staining. (C) Percentage of viable tumour growth from day 0 to day 15 of treatment. Viable tumour volume refers to tumor without necrotic regions (Materials and Methods). Error bars indicate mean ± S.E.M. (D) BIRC6 IHC staining intensity of tumors of control ASO-6w2- and 6w5-treated groups at the end of treatment. (E) Percentage of Ki-67 positive cells as determined by IHC of 6w2- and 6w5-treated tumors at harvest. (F) Representative images of control ASO-6w2- and 6w5-treated PC-3 xenografts using H&E, and IHC staining of BIRC6, cleaved-caspase 3 and Ki-67. 20x magnification.

The therapeutic potential of the dASOs was examined *in vivo*. NOD-SCID mice carrying subcutaneous PC-3 xenografts were treated daily for 15 days with dASOs 6w2, 6w5 or mismatched (MM) ASO (10 mg/kg). Tumor volumes were determined at the end of the treatment; there was no significant difference in total volume between tumors in control and treatment groups (Fig. 4A). However, as revealed by H&E staining, tumors in the dASO-treated groups were found to contain a significantly higher percentage of tumor necrosis compared to the control group (46.67% ± 7.86 and 46.25% ± 8.17 % of necrotic area for 6w2 and 6w5 compared to 19.33% ± 9.49 in the control; mean % of necrotic area ± S.E.M, Fig. 4B). To estimate the viable tumor volume, we used the calculation: total tumor volume x (100% - % of necrotic area). As shown in Figure 4C, mice treated with dASOs 6w2 and 6w5 showed significantly lower viable tumor volume, with percentage of viable tumor volume to control of 61.69% ± 9.30, p = 0.0139 and 58.56% ± 9.14, p = 0.0078, respectively.

The dASO-reduced tumor growth was associated with a significant decrease in intratumoral BIRC6 protein expression in both treatment groups compared to the MM control (p = 0.026 for 6w2, p = 0.006 for 6w5) (Fig. 4D). However, no discernable reduction in the secondary target levels cIAP1 and survivin, was detected via IHC staining, in the tumours under the current treatment regimen. Ki-67 staining showed that the suppressed tumour growth was associated with a significant decrease in the number of proliferating cells in the 6w2 treated group (p = 0.045). 6w5 treatment was also associated with a reduction in the number of proliferating cells although statistical significance was not reached (Fig. 4E, F). No significant increase in cleaved caspase-3 expression was observed in the dASO-treated tumours at harvest (Fig. 4F). The treatment with the dASOs did not induce host toxicity as the weights of the mice were not significantly affected during the course of the treatment (Fig. S6); furthermore, the treated tumors looked pallid compared to the untreated tumors (data not shown). Taken together, the results indicate that treatment with dASOs 6w2 and 6w5 suppressed PC-3 tumor growth *in vivo* without major toxicity to the host.

## DISCUSSION

Despite recent advances in prostate cancer therapy, disease progression is still unavoidable, and treatment resistance remains the major challenge in the management of the disease [8, 32]. It is well accepted that treatment resistance of cancers is largely based on resistance to apoptosis. In particular, upregulation of inhibitors of apoptosis proteins (IAP) is considered to be one of the major mechanisms via which cancer cells can evade cell death [7, 8]. In the present study, we established that elevated expression of BIRC6 protein, a less investigated IAP family member, is correlated with poor prognosis of prostate cancer patients (Fig. 1A-D). This is consistent with our previous study demonstrating that BIRC6 is upregulated in Gleason 6-8 prostate cancers and CRPC [28]. In addition, similar correlations were found for survivin (Fig. S2), an IAP which has been implicated in prostate cancer [11-13]. As such, BIRC6 represents an attractive therapeutic target for prostate cancer.

Here we report the first therapeutic agents developed to target BIRC6 in prostate cancers. dASOs 6w2 and 6w5 simultaneously target BIRC6 and an additional secondary IAP target (cIAP1 or survivin). Both dASOs demonstrated a more rapid protein knockdown of BIRC6 than either cIAP1 or survivin *in vitro* (Fig. S4), suggesting a more time-efficient knockdown of the primary target by dASOs. The stability of protein is another contributing factor that determines the time of protein reduction after dASO treatment. Since cIAP1 and survivin have relatively short half-lives, about 2.8 hours and 30 minutes, respectively [33, 34], their stability is not likely contributing to the delay in protein reduction. Instead, action of dASOs appears to be the major explanation. Secondary targets are expected to be less effectively targeted than BIRC6 due to the presence of mismatched base pairs in the dASOs (Fig. S1).

The very marked growth-inhibitory effects of the 6w2 and 6w5 dASOs on PC-3 and C4-2 cell proliferation (Fig. 2D, E), and on the growth of PC-3 xenografts (Fig. 4), indicate that such dASOs are potentially useful for treatment of advanced prostate cancer, especially since their use did not induce major host toxicity (Fig. S6). It is worth noting that a substantial culture growth inhibition was obtained by treatment with 6w2 or 6w5 alone (Fig. 2D, E). This is in contrast to growth inhibition reported for most IAP antagonists [35]. For instance, targeting cIAP1/2 and/or XIAP by Smac-mimetics alone did not induce cell death in most cancer cell lines, but rather only enhanced apoptosis and cell sensitivity to chemotherapeutics and radiation [9, 36-40]. Likewise, LY2181308, a survivin-targeting ASO (Eli Lilly), and AEG 35156, an XIAP-targeting ASO (Aegera Therapeutics), were shown to effectively induce apoptosis *in vitro* only when combined with gemcitabine (or paclitaxel) and TRAIL, respectively [13, 41]. This highlights the distinctive growth-inhibitory effect that can be obtained by the BIRC6-based, dual IAP-targeting ASOs.

The growth-suppressive effects of the dASOs may be explained by the functional diversity of the primary and secondary IAP targets. BIRC6 has been shown to target pro-apoptotic molecules in the intrinsic apoptotic pathway. In contrast, cIAP1 exerts its anti-apoptotic activity primarily through NFkB-activated survival signaling through the extrinsic apoptotic pathway [42], which was not observed for BIRC6 (Fig. S5). BIRC6 is functionally different from survivin in that it targets precursor and mature forms of caspases 9 and smac for ubiquitin-proteosomal degradation without affecting effector caspases [16]. Survivin, on the other hand, binds to and suppresses the cleavage activities of activated effector caspases 3 and 7 [43]. Accordingly, dual targeting of BIRC6 and cIAP1 or survivin would more effectively induce cancer cell death through acting simultaneously on mutually exclusive pathways.

Various mechanisms appear to play a role in the dASO-induced growth inhibition of the prostate cancer cells. The increase in apoptosis observed in the PC-3 cell cultures is fully expected in view of the reduction in IAP expressions (Fig. 3A-D). Similarly, the accumulation of cells in the G2-M phase (Fig. 3E) is consistent with the roles reported for BIRC6 and survivin in cytokinesis [26] [44]. The suppression of NFkB activation (Fig. 3G) can be explained by a critical role of cIAP1 as an upstream regulator of NFkB [45] and a regulatory role of survivin in NFkB activation [46].

Although both dASOs demonstrated substantial anti-cancer activity *in vivo*, the inhibition of secondary targets is not as obvious as observed *in vitro*. This may be due to, first, detection of protein knockdown (by IHC) was not sensitive enough. Second, tumor cells with both BIRC6 and cIAP1/survivin silenced could have underwent apoptosis during the early phase of treatment, thus, silenced cells were not captured in the current detection window. This may also explain the lack of increased apoptosis observed *in vivo* from cleaved-caspase 3 IHC staining (Fig. 4F).

The use of second-generation ASOs, with 2′-methoxyethyl modifications in their backbone, would greatly improve the treatment efficacy and knock-down efficiency due to their higher tissue half-life and target affinity [47]. Further evaluation of the therapeutic efficacy of dual IAP-targeting ASOs using patient-derived prostate cancer xenograft mouse models of various stages of prostate cancer [48], and in combination with other therapies, appear warranted.

In conclusion, the present study indicates that BIRC6-based dual-IAP targeting ASOs may represent novel therapeutic agents against advanced prostate cancer.

## METHODS

### Materials

Chemicals, solvents and solutions were obtained from Sigma-Aldrich, Oakville, ON, Canada, unless otherwise indicated.

### Antibodies

Anti-BIRC6 (Novus Biologicals, #NB110-40730) [28], anti-survivin (71G4B7) (Cell Signaling, #2808) [49]; anti-XIAP (H-202) (#sc-11426, Santa Cruz Biotechnology, Santa Cruz, CA) [50]. cIAP-1/HIAP-2 antibody (R&D Systems #MAB8181) for IHC, anti-cIAP1 (D5G9) (#7065, Cell Signaling Technology, Danvers, MA) for Western Blotting. The same antibodies were used for immunohistochemistry and Western blotting unless otherwise indicated.

### Cell lines

PC-3 human prostate cancer cell lines were obtained from the American Type Culture Collection (1991, ATCC). C4-2 cells were kindly provided by Dr. L.W.K. Chung (1992, MD Anderson Cancer Center, Houston, Tx). They were maintained as monolayer cultures in RPMI-1640 (Gibco BRL, Gaithersburg, MD) supplemented with 10% fetal bovine serum (FBS). Prior to usage, cells were determined to be mycoplasma free (Mycoplasma Detection Kit, Invitrogen # rep-pt2) and were not authenticated.

### Tissue microarray (TMA) construction and immunohistochemistry (IHC)

Prostate specimens (60 benign prostate samples, 137 primary tumors with no lymph node metastasis, 30 primary tumors with lymph node metastasis, 65 neo-adjuvant treated primary tumors, 67 CRPCs) were obtained from the Vancouver Prostate Centre Tissue Bank following written informed patients’ consent and institutional study approval. All samples had been obtained through radical prostatectomy except the CRPC samples that were obtained through transurethral resection of prostate (TURP). TMAs were constructed as previously described [51]. Immunohistochemical staining using rabbit polyclonal antibody against BIRC6 (NB110-40730, Novus Biological, 1:50), rabbit monoclonal antibody against Survivin (#2808, Cell Signaling, 1:50), monoclonal antibody against cIAP1 (MAB8181, R & D Systems, 1:200) and rabbit polyclonal antibody against XIAP (#sc-11426, Santa Cruz, 1:25) was conducted using a Ventana autostainer (model Discover XT; Ventana Medical System, Tucson, AZ) with an enzyme-labelled biotin-streptavidin system and a solvent-resistant DAB Map kit (Ventana). Descriptively, 0 represents no staining by any tumor cells, 1 represents a faint or focal, questionably present stain, 2 represents a stain of convincing intensity in a minority of cells and 3 a stain of convincing intensity in a majority of cells.

### Dual IAP-targeting ASO design and validation

Dual IAP-targeting ASOs (dASOs) were designed as 20-mers with perfect complementary matches to *BIRC6* mRNA sections and containing no more than 3 base mismatches to the second target mRNA (i.e. cIAP1 or survivin). Sequence alignment to each pair of targeted genes was performed using Clustalw (http://www.genome.jp/tools/clustalw/) and BLAST 2 Sequence in NCBI (http://www.ncbi.nlm.nih.gov/blast/bl2seq/wblast2.cgi) to identify sequences with highest complementarities. ASOs with full phosphorothioate-modified backbone were purchased (Eurofins MWG Operon). The dASO knock-down efficacy of six designed dASOs was tested by determining target protein expression 48 hours after transfection using Western blot analysis. Two dASO candidates (6w2 and 6w5) were selected for further studies: dASO 6w2 (5′CTGCAGCATCATGTGGACT) and dASO 6w5 (5′CAGGTGAAACACTGGGACA). Non-targeting control ASOs: Scramble (Scrb) B control (5′CCTTCCCTGAAGGTTCCTCC), and mismatched (MM) control (5′CAGCAGCAGAGTATTTATCAT). Further information on dASO targeting regions and presence of mismatches to target mRNA are shown in supplementary Figure S1.

### siRNA and ASO transfections

Small interfering RNAs (siRNAs) targeting *cIAP1* (si-cIAP1, siGENOME SMARTpool human BIRC2), *survivin* (si-Surv, siGENOME SMARTpool human BIRC5), *BIRC6* [si-BIRC6, 5′-GUU-UCA-AAG-CAG-GAU-GAU-G-dTdT-3′, [52]] and negative control (siCtrl) siRNAs were purchased from Dharmacon (Cat #M-004390-02-0005, M-003459-03-0005 and D001810-10-05, Chicago, IL). Cells were transfected with siRNA (2 nM for si-survivin and si-cIAP1, 10 nM for si-BIRC6) or ASO (100-200 nM) for 72 hours using oligofectamin reagent (Invitrogen) following the manufacturer's instructions.

### Western blotting

Cell lysates were prepared using cell lysis buffer (1% NP-40, 0.5% sodium deoxycholic acid) supplemented with a protease inhibitor cocktail (Roche, Nutley, NJ). For detection of BIRC6 (528 kDa), 10 μg whole cell lysate was resolved in 5% SDS-polyacrylamide gel and electrotransferred to a PVDF membrane in tris (25 mM), glycine (191.5 mM), methanol (10%), SDS (0.05%) buffer at 40V overnight at 4°C. Membranes were probed with anti-BIRC6 antibody (1:500; Novus Biologicals) at room temperature for 2.5 hours. For detection of cIAP1 and survivin, lysate was resolved in 10% and 15% SDS-polyacrylamide gel, respectively, and electrotransferred to a PVDF membrane in tris (25 mM), glycine (191.5 mM), methanol (10%) buffer at 100V for 1 hour. Membranes were probed with anti-cIAP1 (1:500; Cell Signaling, #7065) and anti-survivin (1:500; Cell Signaling, #2808) antibodies at room temperature for 2.5 hours. Actin or vinculin were used as loading controls and detected on membranes using rabbit anti-actin polyclonal antibody (1:2000; Sigma-Aldrich) or mouse anti-vinculin antibody (1:5000; Sigma-Aldrich).

### Annexin V assay

Apoptosis was detected by fluorescence-activated cell sorter (FACS) analysis with annexin-V conjugated with fluorescein isothiocyanate (Annexin-V-FITC) (Invitrogen) and propidium iodide (PI) staining following the manufacturer's protocol as previously described [28]. Early apoptotic cells were identified as Annexin-V positive, PI negative. Data are presented as means ± SD of triplicate experiments.

### MTS cell viability assay

C4-2 cells (1 × 10^5^) or PC-3 cells (2.5 × 10^4^) were seeded onto 12-well or 24-well culture plates and transfected the next day. MTS (Promega, Madison, MI) was added to wells at 0, 48, 72 and 96 hours after transfection and incubated for 2 hours at 37°C. Aliquots (100 μl) of the culture medium were transferred to a 96-well plate for measuring absorbance at OD490. Triplicate wells were tested per assay and each experiment was repeated twice.

### Cell proliferation assay

PC-3 cells (5 × 10^4^) were seeded onto 12-well plates and transfected with ASOs the next day. Cell numbers were counted at 0, 48, 72, 96 hours after transfection using a TC10™ Automated Cell Counter (Bio-rad Laboratories, Inc, Berkeley, CA). Triplicate wells were tested per assay and the experiment was repeated twice. Results are presented as percentage of untreated control values, mean ± S.D.

### Cell cycle analysis

Cell cycle distribution was determined by flow cytometry of PI-stained cells as previously described [28]. Cells were fixed at 72 hours after transfection. The proportion of cells in G1, S, and G2-M phases of the cell cycle was determined using a FlowJo program (TreeStar Inc, Ashland, OR).4,6-Diamidino-2-phenylindole (DAPI) staining. PC-3 cells were seeded on cover slips in 12 well-plates and transfected with ASO the next day. After 72 hours of transfection, cells were washed twice with PBS and slides were mounted using VECTASHIELD Mounting Medium with DAPI (Vector Laboratories, CA). Cell morphology was examined under a fluorescent microscope (Carl Zeiss, Germany). Cells exhibiting fragmented nuclear bodies were considered to be undergoing apoptosis. A total of 500 cells were counted in five randomly selected fields per sample using a magnification of 400x.

### Dual luciferase reporter assay

PC-3 cells (7×10^3^) were seeded onto 96-well plates and co-transfected the next day with 0.05 μg pGL4.32 [luc2P/NF-kB-RE/Hygro] (# E849A, Promega Corp., Madison, WI), 1 ng pRL-CMV (Renilla) and 100 nM dASOs or 10 nM si-BIRC6 or 2 nM si-cIAP1 using lipofectamine 2000, following the manufacturer's instructions. Cells were incubated with 20 ng/ml TNFα for 5 hours at 37°C for induction of NFκB signalling. Luciferase activity was assessed with a Dual-luciferase reporter assay system (#E1910, Promega) at 48 hours after transfection and measured using a Tecan, Infinite 200Pro microplate reader (Tecan, Männedorf, Switzerland) following the manufacturer's instructions. Transfection efficiency was normalized to Renilla luciferase activity. Fold induction of NFĸB signaling was calculated as average normalized relative light units of induced cells/average normalized relative light units of non-induced cells. Triplicate wells were tested per assay and the experiment was done in duplicate.

### Animal studies

PC-3 cells (1 × 10^6^) were mixed with matrigel and inoculated subcutaneously in both flanks of 6- to 8-weeks-old NOD-SCID mice under isoflurane anesthesia. When tumors reached a volume of 50-70 mm^3^, mice were randomized into 3 groups (n = 12 tumors per group), control ASO, dASO 6w2 and dASO 6w5. The ASOs were administrated to the mice by intraperitoneal injection once daily for 15 consecutive days at a dose of 10 mg/kg. Tumor volume was measured on day 0 and on day 15, the last day of treatment, using the formula: volume = (width)^2^ x length/2. Mice were euthanized on day 15 and tumors fixed for immunohistochemical staining. Percentage of tumor growth represents the change in tumor volume measured on days 1 and 15. Viable tumor volume refers to total tumor volume x (100% - % of necrotic area), where % of necrotic area was determined by microscopic examination of H&E stained sections. Scoring of BIRC6 was determined on a four-point scale as mentioned above. Ki-67 positive cells were counted in 6-8 randomly selected fields (40x magnification) and results are presented as percentage of cells with Ki-67 positive nuclei compared to the total number of cells.

### Statistical analyses

Comparisons of two groups were made using the Student t test. Analyses of correlations between IAP members were performed using a Spearman non-parametric test. Analyses of correlation between BIRC6 expression trend and various prognostic factors were carried out using the Chi square test for trend. Statistical analyses were performed using GraphPad Prism 4.0 (GraphPad). Results with a p<0.05 were considered significant.

## SUPPLEMENTAL MATERIAL AND FIGURES


