# Supplementary material for: The BIRC6 gene as a novel target for therapy of prostate cancer: dual targeting of inhibitors of apoptosis
Source: Oncotarget. 2014 Jul 17;5(16):6896–908. doi: 10.18632/oncotarget.2229 (PMC4196171; doi:10.18632/oncotarget.2229)
Supplement: Supplementary file 1 [file oncotarget-05-6896-s001.pdf]

The *BIRC6* gene as a novel target for therapy of prostate cancer: Dual targeting of Inhibitors of Apoptosis

Supplementary Material

dASO	Primary Target	% match	Secondary target	% match
6w2	BIRC6 mRNA, nt 9299- 9281	19/19 (100%),	BIRC2 transcript variant 2 mRNA, nt 955-937	18/19 (95%)
			BIRC2 transcript variant 1 mRNA., nt 678-660	18/19 (95%)
6w5	BIRC6 mRNA nt 12035-12017	19/19 (100%),	BIRC5 transcript variant 1-3 mRNA, Nt 282-300	16/19 (84%)

Supplementary Figure S1: Primary and secondary targeted regions and percentage of matching nucleotide sequences for the two selected dASOs.

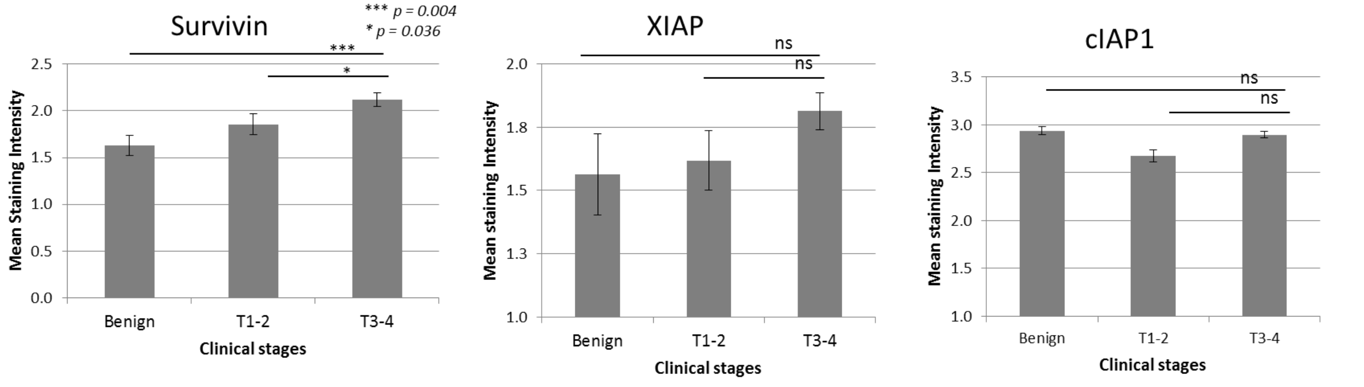

Survivin			
Clinical stages	Benign	T1-2	T3-4
Mean staining	1.63	1.86	2.12
Std. Error	0.11	0.11	0.07
N	32	55	91

XIAP			
Clinical stages	Benign	T1-2	T3-4
Mean staining	1.56	1.62	1.81
Std. Error	0.16	0.12	0.07
N	32	55	91

cIAP1			
Clinical stages	Benign	T1-2	T3-4
Mean staining	2.94	2.67	2.90
Std. Error	0.04	0.06	0.03
N	32	55	86

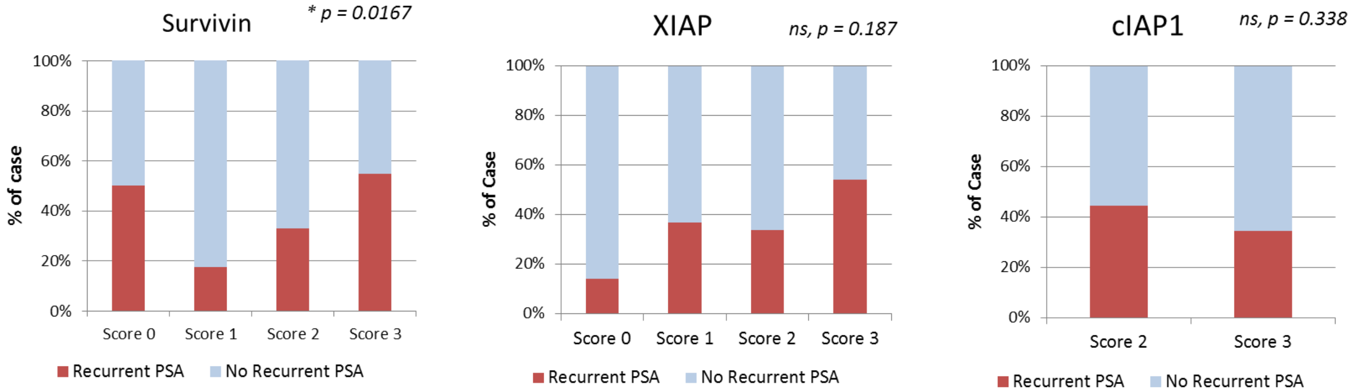

Survivin score	0	1	2	3
N	4	23	70	42

XIAP score	0	1	2	3
N	7	50	62	24

cIAP1 score	2	3
N	27	110

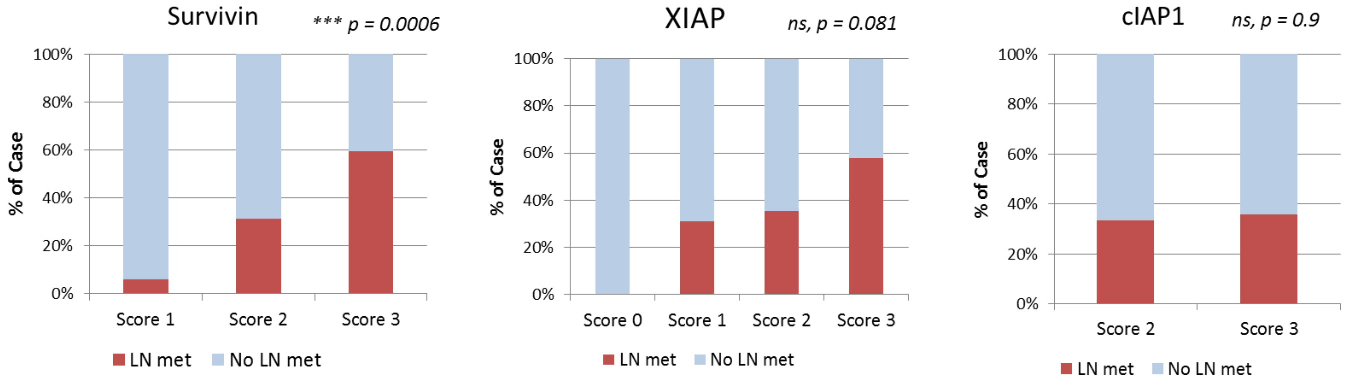

Survivin score	1	2	3
N	17	54	32

XIAP score	0	1	2	3
N	5	30	51	19

cIAP1 score	2	3
N	6	92

Supplementary Figure S2: Association of survivin, XIAP and cIAP1 expressions with clinical status and prognostic features of prostate cancer. Analyses of correlation between BIRC6 expression trend and various prognostic factors were carried out using the Chi square test for trend. Ns, statistically not significant.

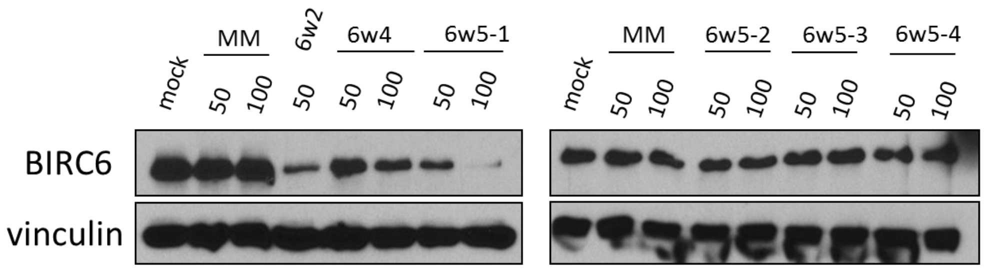

Supplementary Figure S3: Screening of BIRC6 protein knockdown by dual-targeting ASO candidates. PC3 cells were transfected with 2 doses (50 and 100 nM) of indicated dual-targeting ASOs and harvested for Western blotting analysis after 48 hr. The 6w2 and 6w5-1 dASOs in the left panel showed significant BIRC6 protein knockdown and were selected for further studies. Only the 50 nM dose of 6w2 was examined as the 100 nM treatment did not yield enough protein for assay. The 6w5-1 is referred to as 6w5 in subsequent descriptions. MM, mismatched control.

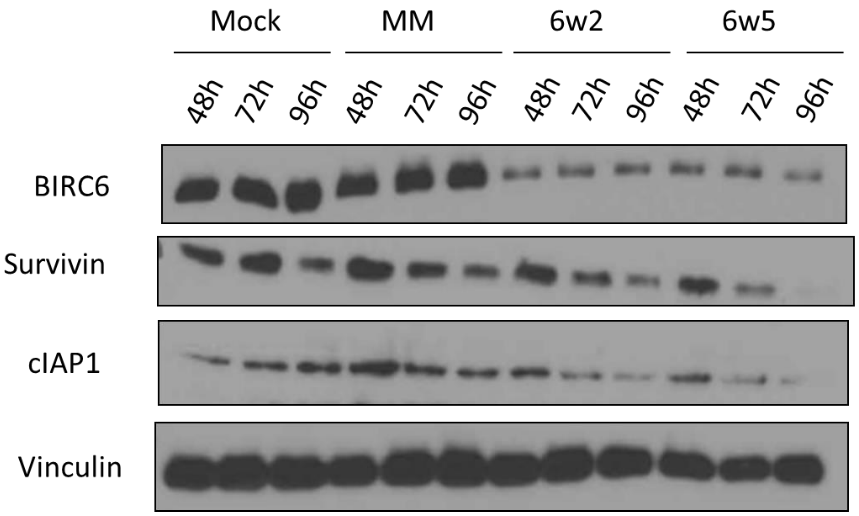

Supplementary Figure S4: Western blotting analysis of BIRC6, cIAP1 and survivin proteins knockdown by dASOs in time course experiment.

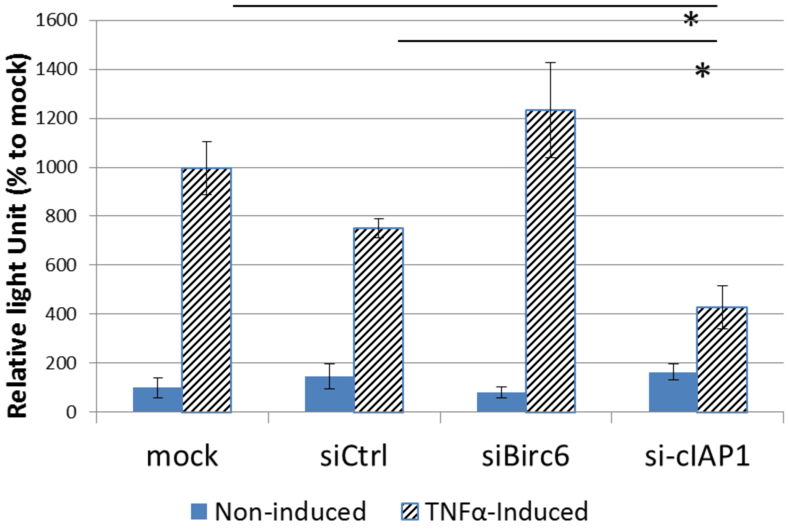

Supplementary Figure S5: Silencing of BIRC6 alone did not affect TNF-α induced NFkB transcription activation.

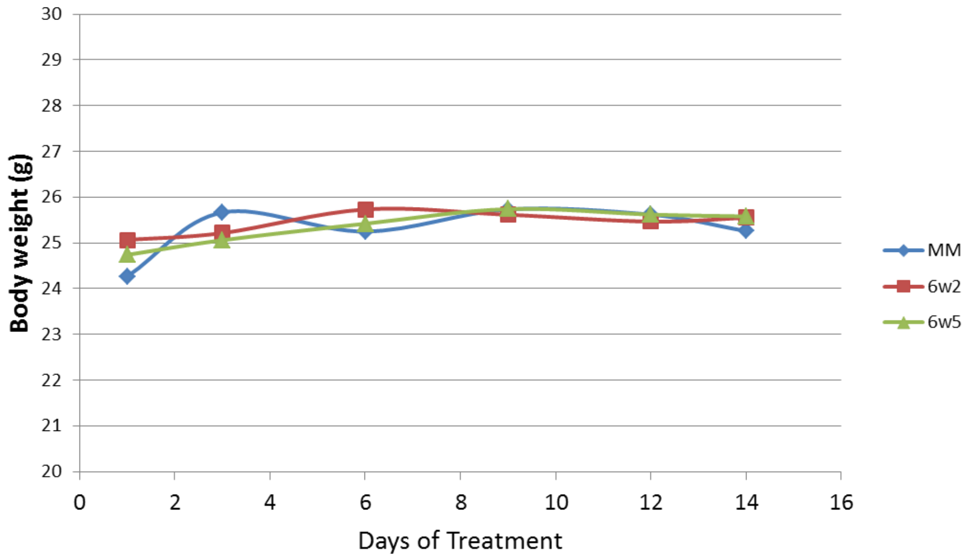

Supplementary Figure S6: Treatment with the dASOs did not affect the weights of the mice.
